# Listeria monocytogenes GlmR Is an Accessory Uridyltransferase Essential for Cytosolic Survival and Virulence

**DOI:** 10.1128/mbio.00073-23

**Published:** 2023-03-20

**Authors:** Daniel A. Pensinger, Kimberly V. Gutierrez, Hans B. Smith, William J. B. Vincent, David S. Stevenson, Katherine A. Black, Krizia M. Perez-Medina, Joseph P. Dillard, Kyu Y. Rhee, Daniel Amador-Noguez, TuAnh N. Huynh, John-Demian Sauer

**Affiliations:** a Department of Medical Microbiology and Immunology, University of Wisconsin—Madison, Madison, Wisconsin, USA; b Microbiology Doctoral Training Program, University of Wisconsin—Madison, Madison, Wisconsin, USA; c Department of Bacteriology, University of Wisconsin—Madison, Madison, Wisconsin, USA; d Weill Cornell Medical College, New York, New York, USA; e Department of Food Science, University of Wisconsin—Madison, Madison, Wisconsin, USA; Universite de Geneve

**Keywords:** GlmR, *Listeria monocytogenes*, cell autonomous defense, cytosolic pathogen, peptidoglycan, uridyltransferase

## Abstract

The cytosol of eukaryotic host cells is an intrinsically hostile environment for bacteria. Understanding how cytosolic pathogens adapt to and survive in the cytosol is critical to developing novel therapeutic interventions against these pathogens. The cytosolic pathogen Listeria monocytogenes requires *glmR* (previously known as *yvcK*), a gene of unknown function, for resistance to cell-wall stress, cytosolic survival, inflammasome avoidance, and, ultimately, virulence *in vivo*. In this study, a genetic suppressor screen revealed that blocking utilization of UDP *N*-acetylglucosamine (UDP-GlcNAc) by a nonessential wall teichoic acid decoration pathway restored resistance to lysozyme and partially restored virulence of Δ*glmR* mutants. In parallel, metabolomic analysis revealed that Δ*glmR* mutants are impaired in the production of UDP-GlcNAc, an essential peptidoglycan and wall teichoic acid (WTA) precursor. We next demonstrated that purified GlmR can directly catalyze the synthesis of UDP-GlcNAc from GlcNAc-1P and UTP, suggesting that it is an accessory uridyltransferase. Biochemical analysis of GlmR orthologues suggests that uridyltransferase activity is conserved. Finally, mutational analysis resulting in a GlmR mutant with impaired catalytic activity demonstrated that uridyltransferase activity was essential to facilitate cell-wall stress responses and virulence *in vivo*. Taken together, these studies indicate that GlmR is an evolutionary conserved accessory uridyltransferase required for cytosolic survival and virulence of L. monocytogenes.

## INTRODUCTION

Bacterial pathogens encounter a variety of stresses throughout the course of infection, including nutritional stresses, redox stresses, and cell-wall stresses. Specifically, the mammalian cytosol restricts the survival and replication of bacteria that are not adapted for that niche (1 to 7). To protect the cytosol, the host utilizes a variety of known and unknown cell autonomous defenses (CADs) that directly target bacterial survival (8, 9). Despite this, canonical cytosolic pathogens such as Listeria monocytogenes can replicate efficiently in this environment. Cytosolic bacterial pathogens have developed adaptions to survive host-imposed stresses in the cytosol (10), acquire necessary nutrients (11), and avoid or subvert innate immune defenses (12, 13). Although many of the adaptations that allow cytosol-adapted pathogens to endure host defenses and stress in the cytosol remain unknown, recent genetic screens have identified some bacterial genes that contribute to cytosolic survival; however, the molecular function of many of these genes remains unknown (7, 14, 15).

A number of virulence factors essential for cytosolic survival of L. monocytogenes, a highly cytosol adapted pathogen, have recently been identified (4, 14, 16, 17). One such protein, GlmR (also known as YvcK or CuvA), is a highly conserved protein found in firmicutes and actinobacteria. In L. monocytogenes and many related organisms, GlmR is dispensable for growth in nutrient-rich media in the absence of stress. In contrast, in multiple organisms, GlmR is essential for growth on limiting gluconeogenic carbon sources and in the presence of stress such as β-lactam antibiotics, host defense proteins such as lysozyme, or survival in the macrophage cytosol (16, 18, 19). Consistent with these functions, L. monocytogenes GlmR protein levels are increased in the presence of cell-wall stress such as the cell-wall-degrading host defense enzyme lysozyme (16). L. monocytogenes GlmR is also necessary for cytosolic survival and replication in host cells (14), and is required for virulence of both L. monocytogenes and Mycobacterium tuberculosis *in vivo* (16, 19, 20). Uniquely, in S. aureus GlmR is predicted to be essential, even in rich media in the absence of cell-wall stress (21). Despite the striking phenotypes of Δ*glmR* mutants in a variety of organisms, molecular function(s) of the protein remain largely unknown in pathogenic bacteria.

How GlmR contributes to cell-wall stress responses and virulence remains largely unknown; however, GlmR was recently described to bind to the essential cell-wall precursor UDP-*N*-acetylglucosamine (UDP-GlcNAc) in *B.subtilis* (22). UDP-GlcNAc is required for the synthesis of peptidoglycan and wall teichoic acid in Firmicutes, as well as arabinogalactan in M. tuberculosis (23 to 25). In B. subtilis, GlmR was found to interact with and regulate the activity of GlmS, one of three highly conserved proteins necessary for UDP-GlcNAc synthesis (26); however, whether this function of GlmR is conserved in related firmicutes or is important for the pathogenesis of organisms like L. monocytogenes remains unknown.

To characterize the function of GlmR in L. monocytogenes, we utilized a genetic suppressor screen to identify second-site mutations that restored lysozyme resistance of the Δ*glmR* mutant. Two independent suppressor mutants that increase pools of available UDP-GlcNAc restored cell-wall stress responses and virulence of Δ*glmR* mutants. In parallel, untargeted metabolomics analysis revealed that Δ*glmR* mutants are deficient in UDP-GlcNAc. We were unable to detect interactions between L. monocytogenes GlmR and its cognate GlmS, as previously reported in B. subtilis, but instead found that purified GlmR, and its orthologues, demonstrate uridyltransferase activity that can catalyze the synthesis UDP-GlcNAc from UTP and *N*-acetylglucosamine-1 phosphate (GlcNAc-1P). Finally, mutational analysis demonstrated that GlmR uridyltransferase activity is necessary to promote cell-wall stress responses and virulence *in vivo*. Together, our data suggest that GlmR is an accessory uridyltransferase that is upregulated to deal with cell-wall stress such as that encountered by L. monocytogenes during cytosolic replication.

## RESULTS

### Inhibition of nonessential decoration of wall teichoic acid with GlcNAc rescues cell-wall stress defects of the Δ*glmR* mutant.

**FIG 1:**
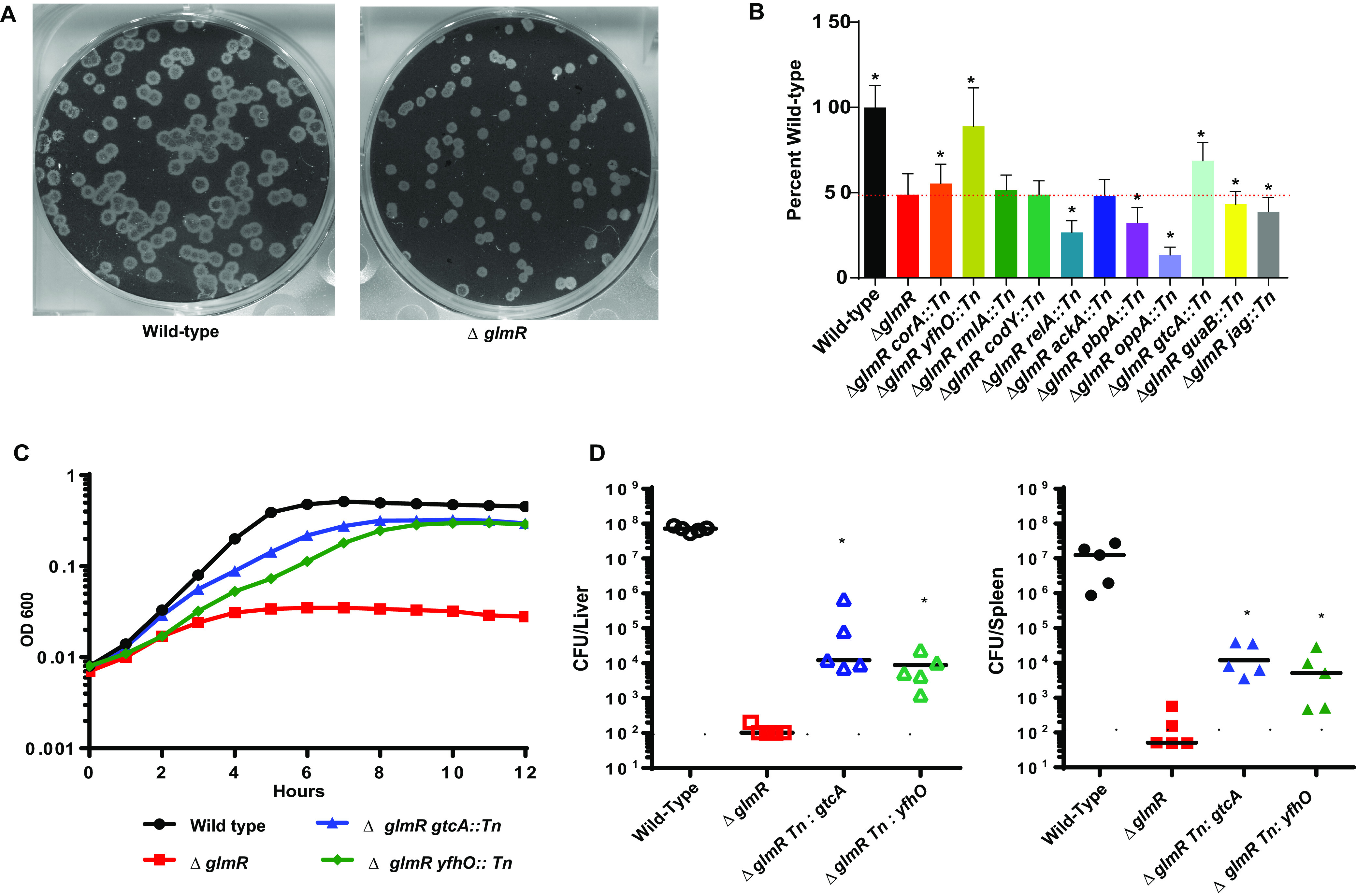
Inhibition of GlcNAc WTA modification suppresses Δ*glmR* mutant phenotypes. (A) Representative image of plaques. (B) Plaque sizes of Δ*glmR* suppressors. Dotted red line indicates Δ*glmR* level. * denotes significant differences from Δ*glmR* by one-way ANOVA (*P* < 0.05). (C) Growth in BHI with 1 mg/mL lysozyme. Graph is representative of greater than 3 biological replicates. (D) C57BL/6 mice were intravenously infected with 1 × 10^5^ bacteria for 48 h, and CFU from spleens (solid) and livers (open) were determined after 48 h. The solid line and dotted line represent the limit of detection for spleen and liver, respectively. Data are representative of two independent experiments. * denotes significant differences by Mann-Whitney test (*P* < 0.05).

L. monocytogenes GlmR is essential for cytosolic survival and virulence, is upregulated in the context of lysozyme stress, and is necessary for resistance to lysozyme (16). To understand how GlmR contributes to cell-wall stress responses and virulence, we performed a lysozyme resistance suppressor selection using a Himar1 mariner-based transposon mutant library in a Δ*glmR* mutant background. Twenty unique transposon insertions disrupting 15 unique genes suppressed the Δ*glmR* mutant’s lysozyme sensitivity (Table 1), and each of these phenotypes was confirmed by transducing the transposons into a new Δ*glmR* mutant. The suppressors represent a diverse set of cellular processes that likely contribute to lysozyme resistance in a variety of ways, including mechanisms that are both generic and GlmR specific. Mutations that generically upregulate stress response pathways may not be useful for understanding GlmR function. Therefore, to prioritize lysozyme suppressor mutants most relevant to the Δ*glmR* mutant virulence defect, we assessed the *ex vivo* virulence of all the transduced Δ*glmR* lysozyme suppressor mutants in a plaque assay. The plaquing assay represents the most complete *ex vivo* assay for virulence of L. monocytogenes requiring cellular invasion, cytosolic survival, intracellular replication, cell-to-cell spread, and secondary vacuole escape (27). In addition to being sensitive to β-lactam antibiotics and lysozyme *in vitro*, Δ*glmR* mutants are unable to form wild-type-sized plaques in fibroblast monolayers (Fig. 1A, B). Only second-site mutations in *yfhO*, *gtcA*, and *corA* statistically significantly rescued the Δ*glmR* plaquing defect (Fig. 1B), while second-site mutations in *relA*, *pbpA*, and *oppA* further inhibited plaquing efficiency of Δ*glmR* mutants. The *yfhO::Tn* and *gtcA::Tn* displayed the most robust suppressor phenotype, so we chose to focus on these mutants for follow-up studies. Both the *yfhO*::*Tn* and *gtcA::Tn* transduced transposons suppress lysozyme sensitivity of a Δ*glmR* mutant, consistent with their identification through the lysozyme suppressor screen (Fig. 1C). Furthermore, the double mutants have no impact on growth in the absence of lysozyme, and the lysozyme sensitivity of the suppressor mutants can be restored by expression of *yfhO* or *gtcA* in *trans*, respectively (Fig. S1). In L. monocytogenes 1/2a strains, both YfhO and GtcA are required for modification of the wall teichoic acid (WTA) repeating ribitol subunits with *N*-acetylglucosamine (GlcNAc) derived from UDP-GlcNAc (15, 28, 29). We confirmed that the Δ*glmR gtcA::Tn* double mutant is defective for GlcNAc WTA decoration based on loss of wheat germ agglutinin staining (Fig. S2 and Text S1). Finally, disruption of *gtcA* or *yfhO* in a Δ*glmR* mutant partially restores virulence in a murine model of disseminated listeriosis (Fig. 1D). Taken together, these data suggest that elimination of nonessential decoration of WTA with GlcNAc increases available pools of UDP-GlcNAc, which can rescue Δ*glmR* mutant lysozyme sensitivity and virulence *ex vivo* and *in vivo*.

**TABLE 1:** Δ*glmR* suppressor mutants^*a*^

Role	lmo number	Name	Function	Number of hits	Number of insertions
WTA modification	1079	*yfhO*	WTA glycosylation	9	2
	1081	*rmlA*	Glucose-1-phosphate thymidyl transferase	1	1
	2549	*gtcA*	WTA glycosylation	1	1
GTP synthesis and metabolic stress response	1096	*guaA*	GMP synthase	1	1
	1280	*codY*	Nutrient response regulator	1	1
	1523	*relA*	ppGpp synthase/reductase	4	2
	2753	*guaB*	Inosine 5′-monophosphate dehydrogenase	6	2
RNA binding	2853	*Jag*	Sporulation-related RNA binding protein	8	1
Transport	1064	*corA*	Mg transport	4	2
	2195	*oppB*	Oligopeptide ABC transporter	9	1
	2196	*oppA*	Oligopeptide ABC transporter	15	2
Acetate metabolism	1581	*ackA*	Acetate kinase	39	1
Peptidoglycan synthesis	1892	*pbpA*	High mol wt penicillin binding protein	1	1

a A Himar 1 transposon mutant library in a Δ*glmR* background was passaged through lysozyme selection. Transposon insertions were identified by sequencing and diagnostic PCR, transduced into a fresh Δ*glmR* background, and reconfirmed. Listed are the identified genes, general role they belong to, the number of hits identified in the selection, and the number of unique insertions.

10.1128/mbio.00073-23.2 FIG S1 Complementation of Δ*glmR* mutant’s yfhO and gtcA. (A) Growth of Δ*glmR* Tn *yfhO* and complementation strain in BHI over 12 h at 37°C. Graph is representative of greater than 3 biological replicates. (B) Growth of Δ*glmR* Tn *gtcA* and complementation strain in BHI over 12 h at 37°C. Graph is representative of greater than 3 biological replicates. (C) Transcomplementation of growth in BHI with 1mg/mL lysozyme over 12 h at 37°C. Graph is representative of greater than 3 biological replicates. (D) Transcomplementation growth in BHI with 1mg/mL lysozyme over 12 h at 37°C. Graph is representative of greater than 3 biological replicates. Download FIG S1, EPS file, 4.6 MB. Copyright © 2023 Pensinger et al. 2023 Pensinger et al. https://creativecommons.org/licenses/by/4.0/ This content is distributed under the terms of the Creative Commons Attribution 4.0 International license.

10.1128/mbio.00073-23.3 FIG S2 GtcA is functionally inactivated by a Tn insertion. Wild-type, *glmR*, and *glmR gtcA::Tn* strains were imaged and assessed for their ability to bind WGA (red). Download FIG S2, EPS file, 6.2 MB. Copyright © 2023 Pensinger et al. 2023 Pensinger et al. https://creativecommons.org/licenses/by/4.0/ This content is distributed under the terms of the Creative Commons Attribution 4.0 International license.

### Δ*glmR* mutants have depleted pools of UDP-GlcNAc.

**FIG 2:**
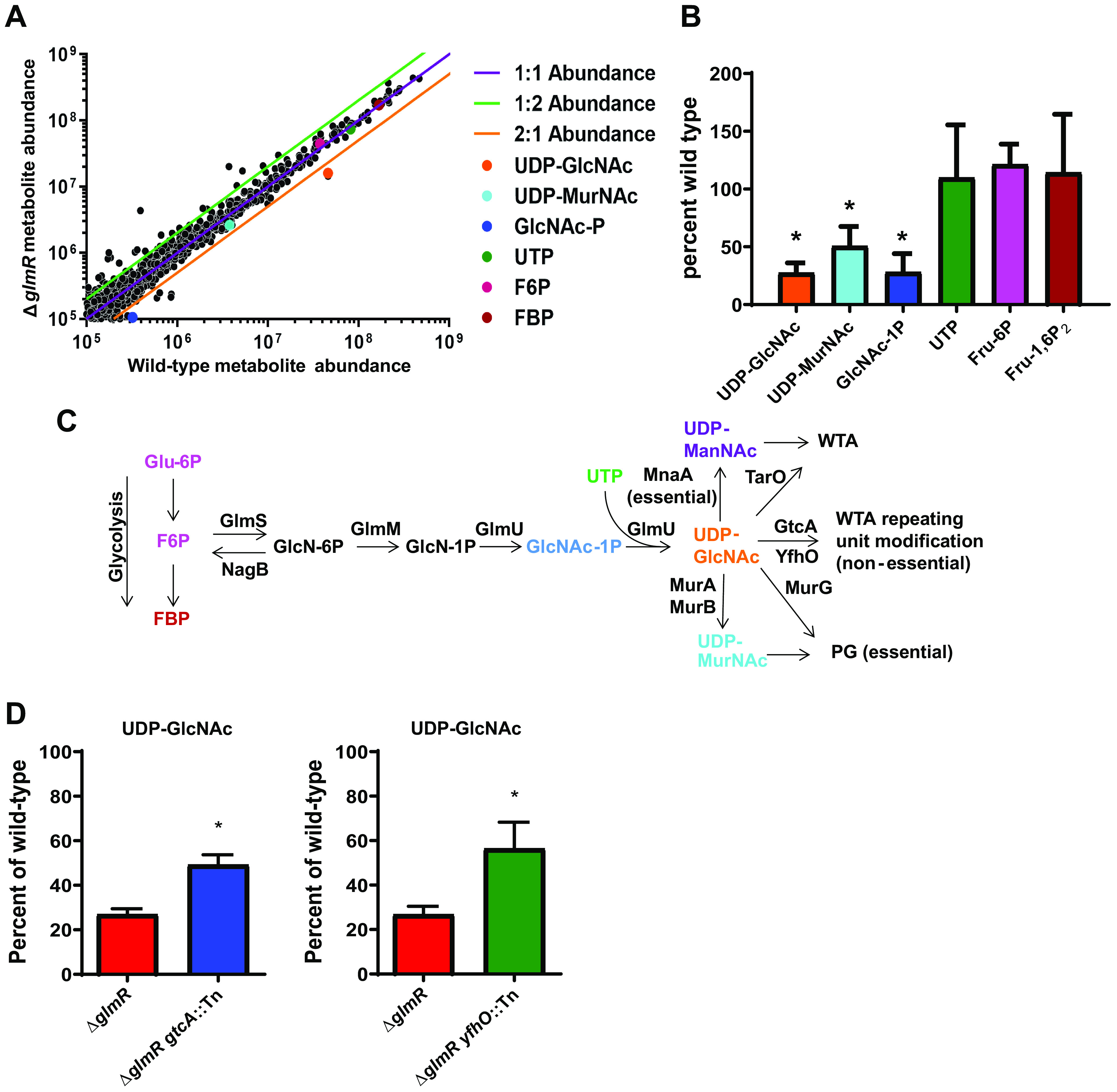
Δ*glmR* mutants are impaired in the production of GlmSMU pathway metabolites. (A) Scatterplot of putative KEGG-identified ions averaged across 4 biological replicates. (B) Quantification of selected metabolites in the Δ*glmR* mutant relative to wild type across 4 biological replicates. * denotes significant differences from wild type by Student's *t* test (*P* < 0.05). (C) UDP-GlcNAc synthesis and utilization pathway. (D) Quantification of selected metabolites in Δ*glmR* suppressor mutants across 3 biological replicates. * denotes significant differences from Δ*glmR* by Student's *t* test (*P* < 0.05).

Loss of GlcNAc decoration of the WTA restored lysozyme resistance and partial virulence to Δ*glmR*-deficient mutants; therefore, we hypothesized that Δ*glmR* mutants may have metabolic defects leading to decreased UDP-GlcNAc synthesis. To test this hypothesis, we utilized untargeted metabolomics to identify differentially abundant metabolites in Δ*glmR* mutants relative to wild-type (WT) L. monocytogenes. After growth in modified *Listeria* synthetic media (LSM), metabolites were extracted and untargeted LC-MS was performed. Subsequent analysis using MAVEN software resulted in 1,073 putative metabolites assigned identities according to their *m/z* and mapped to the Kyoto Encyclopedia of Genes and Genomes (KEGG) (30). Importantly, although not every metabolite was validated by MS-MS or control standards, 37 putative metabolites were identified with >2-fold differences between wild type and the Δ*glmR* mutant across three biological replicates (Fig. 2A, Table S1). The relatively small number of differentially abundant metabolites suggests that GlmR does not have a global regulatory function, at least under the growth conditions tested. Consistent with our hypothesis, UDP-GlcNAc was among the most differentially abundant metabolites in Δ*glmR* mutants compared to wild-type L. monocytogenes. UDP-GlcNAc levels were reduced >3-fold in the Δ*glmR* mutant (Fig. 2B) relative to wild-type controls, consistent with the hypothesis from the suppressor screen that UDP-GlcNAc metabolism is disrupted in the Δ*glmR* mutant. UDP-*N*-acetyl-muramic acid (UDP-MurNAc), another peptidoglycan precursor downstream of UDP-GlcNAc (Fig. 2B, C), was similarly decreased in the Δ*glmR* mutant (~50% of wild type). UDP-GlcNAc is synthesized by the GlmSMU pathway that converts fructose-6-phosphate into UDP-GlcNAc through a four-step enzymatic process (Fig. 2C). Upstream of UDP-GlcNAc in the GlmSMU pathway, *N*-acetylglucosamine-1 phosphate (GlcNAc-1P) levels were also significantly reduced in the Δ*glmR* mutant; however, UTP levels were unchanged (Fig. 2B, C). We analyzed other metabolites in the GlmSMU pathway but were unable to observe the GlmSMU pathway intermediates glucosamine-1 phosphate (GlcN-1P) and glucosamine-6 phosphate (GlcN-6P) (Fig. 2B, C) via our mass spectrometry (MS) method even when running purified standards. Finally, levels of the glycolytic intermediates fructose-6-phosphate (F6P) and fructose-1,6-bisphosphate (FBP) were unchanged in the Δ*glmR* mutant, suggesting that deficits in muropeptide precursors are due specifically to alterations in the UDP-GlcNAc synthesis pathway and not in a more central metabolic pathway (Fig. 2B, Fig. 2C). Consistent with the model that blocking a nonessential UDP-GlcNAc utilizing pathway increases available UDP-GlcNAc for essential peptidoglycan (PG) or WTA synthesis, targeted metabolomics analysis of both the Δ*glmR gtcA::Tn* and the Δ*glmR yfhO::Tn* suppressor mutants with specific standards for UDP-GlcNAc demonstrated significant rescue of UDP-GlcNAc levels, though not all the way back to wild-type levels (Fig. 2D). Incomplete restoration of UDP-GlcNAc levels in these suppressor mutants could explain the partial complementation phenotype for the suppressors in the context of lysozyme sensitivity and virulence *in vivo* (Fig. 1C, D). Taken together, these data demonstrate that Δ*glmR* mutants have reduced levels of UDP-GlcNAc and suggest that restoration of UDP-GlcNAc pools restores cell-wall stress responses and virulence *in vivo.*

10.1128/mbio.00073-23.9 TABLE S1 Putative KEGG-identified differential metabolites. Putative KEGG identified metabolites with greater than 2-fold abundance differential between wild type and Δ*glmR*, their median *m/z*, abundance in wild type and the *glmR* mutant, and ratio between the two are listed. Download Table S1, DOCX file, 0.02 MB. Copyright © 2023 Pensinger et al. 2023 Pensinger et al. https://creativecommons.org/licenses/by/4.0/ This content is distributed under the terms of the Creative Commons Attribution 4.0 International license.

### GlmR is an accessory uridyltransferase.

Two recent studies suggested that GlmR’s function in B. subtilis is to enhance the activity of GlmS through direct GlmR–GlmS interactions, thus increasing levels of UDP-GlcNAc (Fig. 2C). These studies utilized bacterial two-hybrid assays to demonstrate a direct interaction between B. subtilis GlmR and GlmS (26), and a subsequent study demonstrated that this interaction modulates GlmS activity (31). To determine if GlmR-GlmS interactions are conserved in L. monocytogenes, we expressed both B. subtilis and L. monocytogenes GlmS and GlmR constructs in the BACTH bacterial two-hybrid system. Each protein was expressed independently as both N- and C-terminal fusions to both T18 and T25. Four replicates of the blue-white assay were performed due to variability in the system from a known thresholding effect (32), and quantitative β-galactosidase assays were performed in triplicate. As predicted based on their crystal structures, GlmS (33) and GlmR (PDB 2Q7X and 1HZB) from both B. subtilis and L. monocytogenes homodimerized, demonstrating that the constructs were expressed and functional (Fig. S3). Positive but inconsistent interactions between B. subtilis GlmR and GlmS were observed as previously reported for one set of B. subtilis fusion proteins (Fig. S4) (26); however, no combination of L. monocytogenes GlmR and GlmS produced an interaction except those for which there was also activity observed in the empty vector controls (Fig. S5). Taken together, these data suggest that GlmR regulation of GlmS through protein–protein interactions may not be evolutionarily conserved among GlmR homologues and that GlmR may function to regulate UDP-GlcNAc levels by a novel mechanism in L. monocytogenes.

10.1128/mbio.00073-23.4 FIG S3 GlmS and GlmR form homodimers. (A and B) Bacterial 2-hybrid strains were plated on X-Gal and incubated for 24 h at 30°C in biological quadruplicate. Download FIG S3, PDF file, 3.8 MB. Copyright © 2023 Pensinger et al. 2023 Pensinger et al. https://creativecommons.org/licenses/by/4.0/ This content is distributed under the terms of the Creative Commons Attribution 4.0 International license.

10.1128/mbio.00073-23.5 FIG S4 B. subtilis GlmR interaction with GlmS. (A and B) Bacterial 2-hybrid strains were plated on X-Gal and incubated for 24 h at 30°C in biological quadruplicate. Bacterial 2-hybrid cultures were lysed and assayed for β-galactosidase activity in biological triplicate. Activity is normalized to the Zip positive control. The dotted red line indicates 10% of the Zip value. Strains are identified by a number and listed below. Control strains are green, GlmS-GlmR interaction test strains are red, and GlmS or GlmR homodimer strains are gold. Download FIG S4, PDF file, 6.1 MB. Copyright © 2023 Pensinger et al. 2023 Pensinger et al. https://creativecommons.org/licenses/by/4.0/ This content is distributed under the terms of the Creative Commons Attribution 4.0 International license.

10.1128/mbio.00073-23.6 FIG S5 L. monocytogenes GlmR does not interact with GlmS. (A and B) Bacterial 2-hybrid strains were plated on X-Gal and incubated for 24 h at 30°C in biological quadruplicate. Bacterial 2-hybrid cultures were lysed and assayed for β-galactosidase activity in biological triplicate. Activity is normalized to the Zip positive control. The dotted red line indicates 10% of the Zip value. Strains are identified by a number and listed below. Control strains are green, GlmS-GlmR interaction test strains are red, and GlmS or GlmR homodimer strains are gold. Download FIG S5, PDF file, 7.5 MB. Copyright © 2023 Pensinger et al. 2023 Pensinger et al. https://creativecommons.org/licenses/by/4.0/ This content is distributed under the terms of the Creative Commons Attribution 4.0 International license.

**FIG 3:**
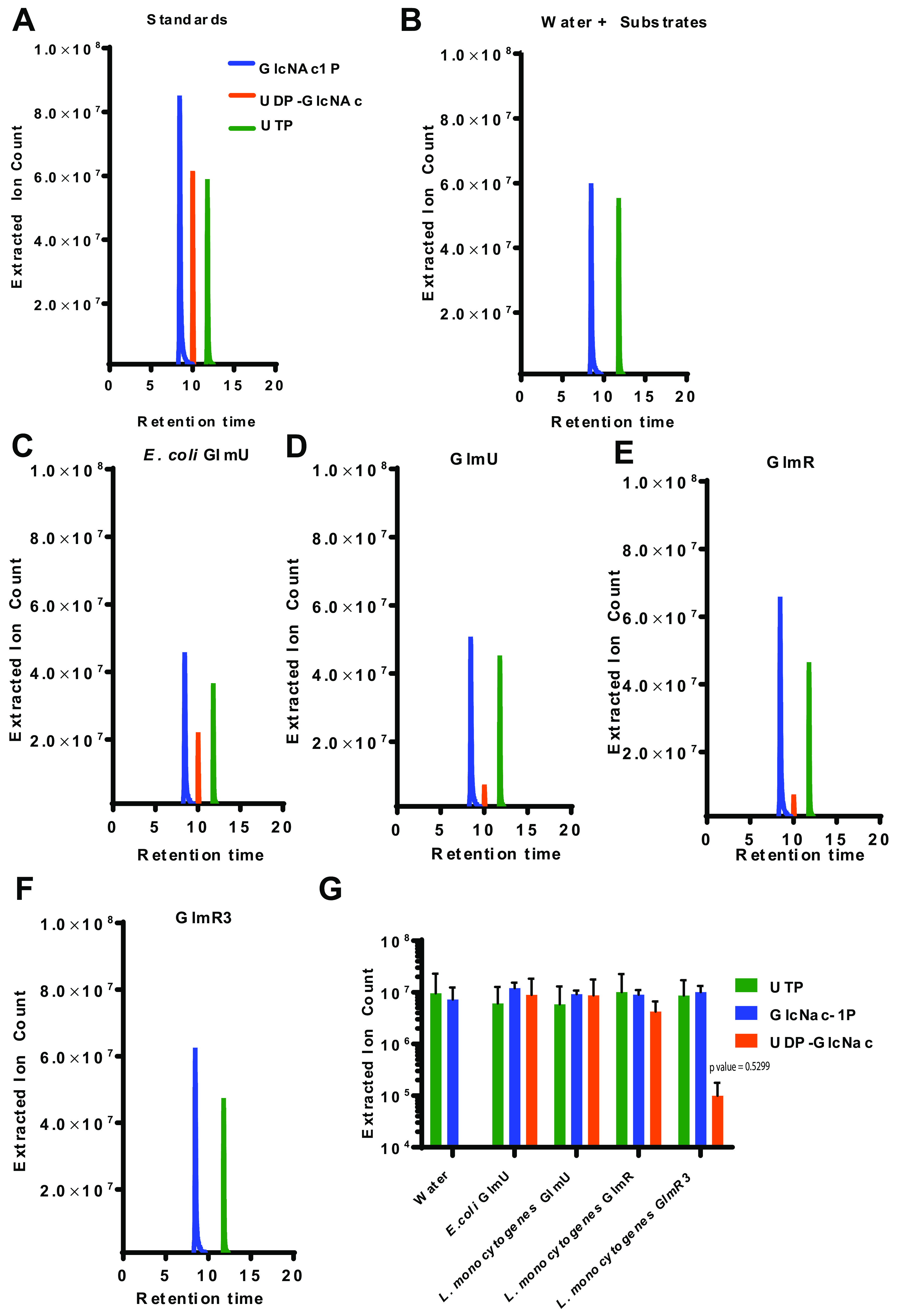
GlmR catalyzes the production of UDP-GlcNAc. (A to F) HPLC-MS analysis of reactions with 100 μM substrates alone or in combination with 1 μM purified GlmU or GlmR as indicated. Extracted ion counts for the relevant metabolites are indicated based on purified standards (GlcNAc-1P blue, UTP green, UDP-GlcNAc orange). (G) Quantification of selected metabolites (GlcNAc-1P blue, UTP green, UDP-GlcNAc orange) from reactions with 100 μM substrates alone or in combination with water, 1 μM *E.coli* GlmU, GlmU, GlmR, and GlmR3. Assays were performed in triplicate. *P* > 0.05.

A distant homologue of GlmR is CofD, a 2-phospho-l-lactate transferase involved in the synthesis of Coenzyme F420 in actinobacteria (34). This homology to a catalytic protein suggests that perhaps GlmR has direct enzymatic activity, perhaps as an accessory enzyme in muropeptide biosynthesis. In multiple Gram-positive pathogens, MurZ is an accessory enzyme involved in muropeptide synthesis that is upregulated in the context of cell-wall stress (35). We had previously demonstrated that GlmR protein levels are similarly increased in the presence of lysozyme (16), leading to the hypothesis that GlmR could be an accessory enzyme functioning to increase pools of UDP-GlcNAc in the context of cell-wall stress. To test this hypothesis, we cloned and purified GlmR from L. monocytogenes and assessed its potential enzymatic activity in the last two steps of the canonical GlmSMU pathway normally catalyzed by GlmU to produce UDP-GlcNAc (Fig. 2C). GlmU is a bifunctional enzyme that contains both acetytransferase and uridyltransferase activity. Using mass spectrometry to assess the results of each reaction, we found that GlmR catalyzed the synthesis of UDP-GlcNAc from GlcNAc-1P and UTP (Fig. 3), similar to both commercially purchased Escherichia coli GlmU as well as L. monocytogenes GlmU that we expressed and purified (Fig. 3). Importantly, no UDP-GlcNAc was detectable with substrates UTP and GlcNAc-1P alone, indicating that catalysis required either the GlmU or GlmR protein (Fig. 3). In contrast, we detected no acetyltransferase activity associated with GlmR, suggesting that GlmR is not a dual-functional enzyme like GlmU and further demonstrating that the GlmR activity observed was not an artifact of accidental copurification of GlmU (Fig. S6A). Importantly, in these reactions, the substrate GlcN-1P is not detectable via our MS method, consistent with our inability to detect this metabolite in our untargeted MS method (Fig. 2). Finally, the absence of UDP-GlcNAc in a GlmR reaction mixture lacking GlcNAc-1P and UTP as the substrates or after the protein was heated excludes the possibility of UDP-GlcNAc being a copurified artifact with GlmR (Fig. S6B). Taken together, these data suggest that GlmR can act as a uridyltransferase enzyme to directly facilitate increased production of UDP-GlcNAc in response to cell-wall stress.

10.1128/mbio.00073-23.7 FIG S6 L. monocytogenes GlmR lacks acetyltransferase activity. HPLC-MS analysis of reactions with 100μM substrates alone (GlcNAc-1P and Acetyl CoA) or in combination with 1μM GlmU or GlmR. Peaks for the relevant metabolites are indicated (Acetyl-CoA black, GlcNAc-1P blue, UDP-GlcNAc orange). (B) Quantification of selected metabolites (GlcNAc-1P blue, UTP green, UDP-GlcNAc orange) from reactions with 100μM substrates (GlcNAc-1P and UTP) alone or in combination with water, 1μM heat-inactivated (HI) GlmR, or heat-inactivated (HI) GlmU. Assays were performed in triplicate. Download FIG S6, EPS file, 0.7 MB. Copyright © 2023 Pensinger et al. 2023 Pensinger et al. https://creativecommons.org/licenses/by/4.0/ This content is distributed under the terms of the Creative Commons Attribution 4.0 International license.

### GlmR uridyltransferase activity is conserved.

**FIG 4:**
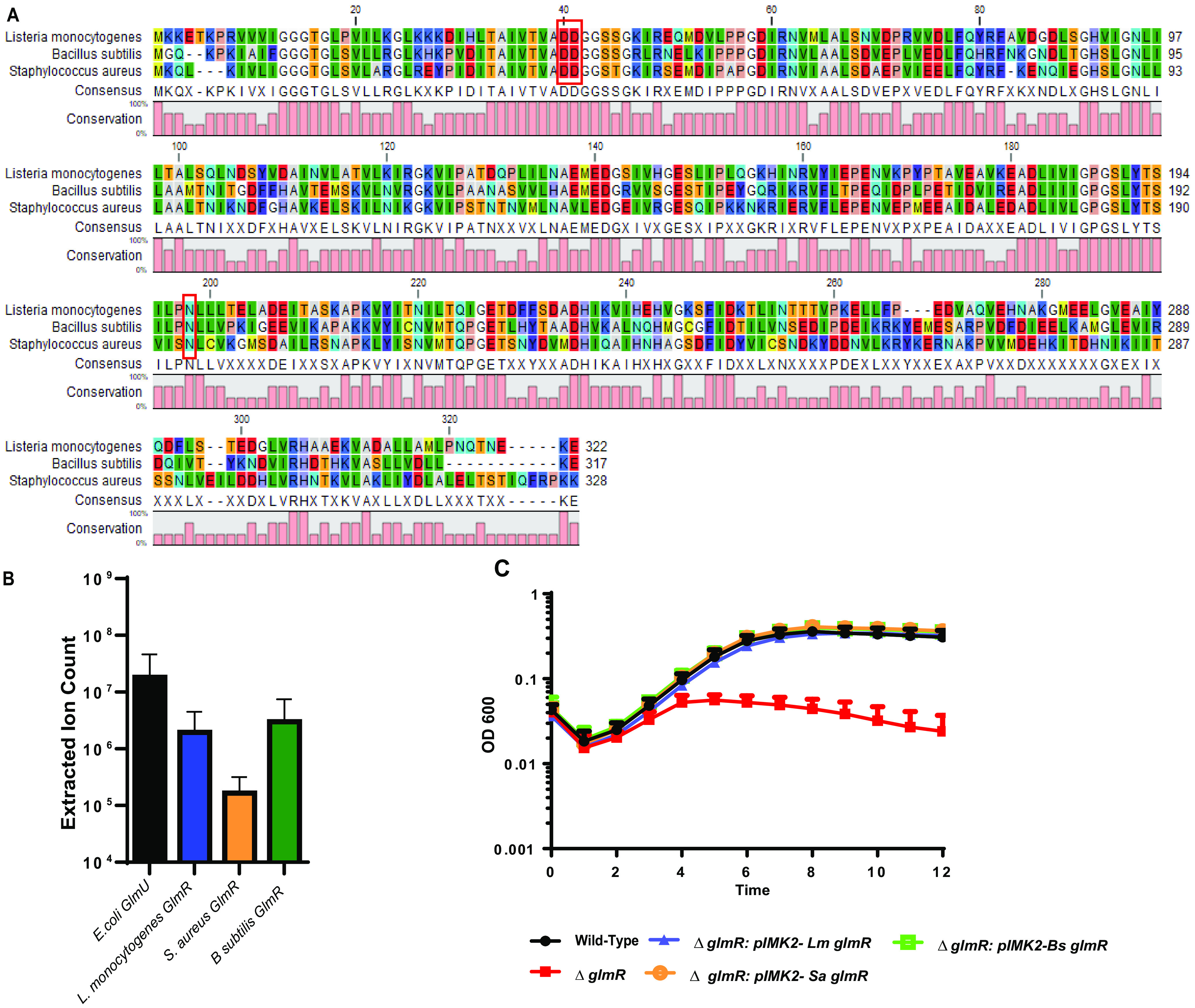
GlmR uridyltransferase function is conserved in S. aureus. (A) GlmR homologues aligned using CLC Sequence Viewer 8.0. Red boxes outline the predicted GlmR catalytic sites. (B) Analysis of uridyltransferase activity of E. coli GlmU and purified GlmR homologues by HPLC-MS. No significant differences by ANOVA. (C) Transcomplementation of growth in BHI with 1 mg/mL lysozyme over 12 h at 37°C. Graph is representative of greater than 3 biological replicates.

GlmR is the second gene of a highly conserved operon of three genes found in firmicutes and actinobacteria. In S. aureus, the GlmR homologue YvcK is predicted to be essential (21), while in *B.subtilis* it is found to be important for synthesis of UDP-GlcNAc (18, 22). The S. aureus and B. subtilis GlmR homologues exhibit high homology to L. monocytogenes GlmR, with 46% identity, 69% similarity and 47% identity, 70% similarity, respectively, and are best conserved near the putative N-terminal active site (Fig. 4A). To determine whether GlmR uridyltransferase enzymatic function is conserved among firmicute homologues, we cloned and purified GlmR from S. aureus and B. subtilis and assessed enzymatic activity. Each protein exhibited uridyltransferase activity similar to L. monocytogenes GlmR (Fig. 4B). To test for functional conservation of GlmR function *in vivo*, we complemented the L. monocytogenes Δ*glmR* mutant with codon-optimized *glmR* homologues from S. aureus and B. subtilis. As hypothesized based on their conserved enzymatic activity, both the S. aureus *and B.subtilis* homologues rescued lysozyme sensitivity of a L. monocytogenes Δ*glmR* mutant when expressed in *trans* (Fig. 4C). Taken together, these data suggest that the uridyltransferase enzymatic function of GlmR is conserved in diverse firmicutes, including both pathogens and nonpathogens.

### GlmR uridyltransferase activity is required for cell-wall stress responses and virulence *in vivo*.

**FIG 5:**
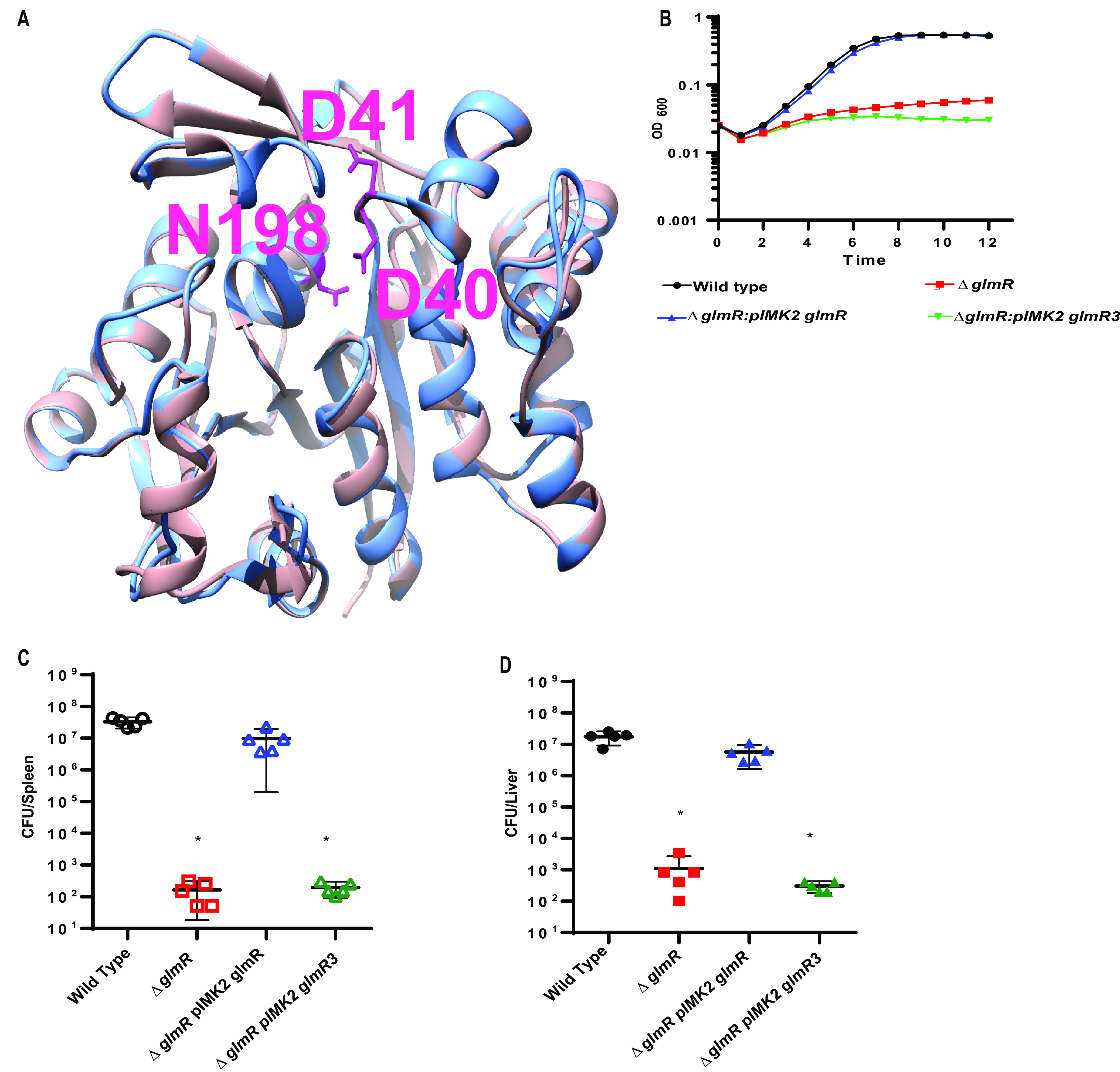
GlmR uridyltransferase activity necessary for virulence. (A) Structural modeling of Lm GlmR. Structural overlay of Bacillus halodurans YvcK (salmon color; later renamed GlmR, PDB 2O2Z) and L. monocytogenes GlmR (light blue), generated by Phyre2 using 2O2Z as a template. Mutations made in the predicted catalytic site are highlighted (hot pink): D41, D40, N198 (from top, clockwise). (B) Growth of WT, Δ*glmR*, Δ*glmR:pIMK2 GlmR*, and Δ*glmR:: pIMK2 GlmR3* in BHI with 1 mg/mL of lysozyme over 12 h at 37°C. Graph is representative of greater than 3 biological replicates. (C) C57/Bl6 mice were infected intravenously with 1 × 10^5^ wild type (black circles), Δ*glmR* mutants (red squares), Δ*glmR:GlmR* mutants (blue triangles), and Δ*glmR::GlmR*3 (green triangles) *in vivo*. Spleens (open) were harvested 48 h postinfection homogenized and plated for CFU. (D) C57/Bl6 mice were infected intravenously with 1 × 10^5^ wild type (black circles), Δ*glmR* mutants (red squares), Δ*glmR::GlmR* mutants (blue triangles), and Δ*glmR::GlmR*3 (green triangles) *in vivo*. Livers (solid) were harvested 48 h postinfection homogenized and plated for CFU. The median (solid bar) and limit of detection (dotted line) for each experiment are indicated. Data are representative of two independent experiments with 5 mice each. * indicates statistical significance by Mann-Whitney test (*P* < 0.05).

Our data suggest that GlmR can act as an accessory uridyltransferase; however, whether this activity is required for cell-wall stress and virulence is unknown. To determine if GlmR uridyltransferase activity is important for L. monocytogenes lysozyme resistance and virulence, we aimed to create a catalytically inactive GlmR. The amino acid sequence of L. monocytogenes GlmR is highly similar to the Bacillus halodurans GlmR homolog (~47% sequence identity), for which the crystal structure (PDB 2O2Z) has been solved. Based on this similarity, we used Phyre2 to generate an L. monocytogenes GlmR structural model, using the 2O2Z structure as a template, and found the two structures to be superimposable (Fig. 5A). We then overlaid the L. monocytogenes GlmR structural model on the N-terminal uridyltransferase domain of *Haemophillus influenzae* GlmU (2V0I), which is structurally and biochemically well characterized (36). This analysis revealed several similar structural elements between L. monocytogenes GlmR and H. influenzae GlmU. For instance, both structures harbor a core set of seven β-sheets sandwiched by α-helices. In the H. influenzae GlmU uridyltransferase active site, residues K25, Q76, and D105 coordinate UTP binding and are absolutely required for enzymatic activity. In this proximity in the GlmR model, we identified D40, D41, and N198 residues that are highly conserved among GlmR homologs (Fig. 4A) and predicted that they are important for catalytic activity. This predicted active site is distinct from the site demonstrated to bind UDP-GlcNAc in *B. subitilis* GlmR, where Y265 and R301 are essential for UDP-GlcNAc binding (31). Notably, Y265 is absent in L. monocytogenes GlmR (Fig. 4A).

To test the hypothesis that uridyltransferase activity is necessary for virulence, we created a D40A, D41A, N198A mutant GlmR (GlmR3), purified the mutant protein, and assessed uridyltransferase activity. Activity of the GlmR3 mutant was ~100-fold reduced in an *in vitro* biochemical assay compared to wild-type GlmR (Fig. 3F, G). Complementation of a Δ*glmR* mutant with *glmR3* was unable to rescue lysozyme sensitivity (Fig. 5B) despite equal or even increased levels of expression compared to the wild-type GlmR complement (Fig. S7). Finally, to test the hypothesis that uridyltransferase activity is important for virulence, we infected mice and quantified bacterial burdens in an *in vivo* model of disseminated listeriosis. In contrast to complementation with wild-type GlmR, the GlmR3 mutant was unable to rescue the virulence defect of the Δ*glmR* mutant (Fig. 5C, D). Taken together, these data suggest that the uridyltransferase activity of GlmR is essential for mediating cell-wall stress responses during infection to facilitate virulence of L. monocytogenes.

10.1128/mbio.00073-23.8 FIG S7 GlmR3 equal or increased expression to WTX GlmR. Expression of GlmR in WT, Δ*glmR*, Δ*glmR:glmR*, and Δ*glmR:glmR3* at midlog in BHI with 250 μg/mL lysozyme. Sequences of the L. monocytogenes expression constructs have been sequenced multiple times to ensure correct sequence. Western blot is a representative of multiple replicates where we reproducibly see the shift in size from the construct expressed in L. monocytogenes but not from the recombinant protein. Download FIG S7, EPS file, 0.6 MB. Copyright © 2023 Pensinger et al. 2023 Pensinger et al. https://creativecommons.org/licenses/by/4.0/ This content is distributed under the terms of the Creative Commons Attribution 4.0 International license.

## DISCUSSION

GlmR is a highly conserved protein that is essential for virulence in L. monocytogenes and M. tuberculosis, but whose function remains largely unknown (16, 19, 20). In this study, we discovered that GlmR has conserved uridyltransferase activity that facilitates cell-wall stress responses during infection. Our findings are also consistent with a recent study utilizing *trans*-Cinnamaldehyde (t-Cin) hypersensitive L. monocytogenes *glmR*:Himar1 mutants, which identified suppressor mutations in genes involved in the biosynthesis of UDP-GlcNAc (37). When the *glmR*:Himar1 mutant was engineered to overexpress *glmU*, growth in t-Cin was fully restored, whereas overexpression of *glmS* or *glmM* only partially restored resistance to *t*-Cin, further supporting the idea that GlmR is involved in the biosynthesis of UDP-GlcNAc and that the terminal step of the canonical GlmSMU pathway is rate limiting (23, 37). Deciphering the activities of proteins of unknown function, such as GlmR, is a major challenge not only in microbial pathogenesis but in biology at large. Indeed, 25% of predicted biochemical reactions do not have an assigned enzyme, suggesting that many proteins of unknown function have enzymatic activity (38, 39). Recent metabolomics approaches such as activity-based metabolomics have shown great promise in identifying these functions (39, 40). Combining parallel screening approaches such as genetics, transcriptomics, proteomics, and metabolomics generates targeted hypotheses about the roles of proteins of unknown function in physiological processes. In this study, an untargeted metabolomics approach combined with a classical bacterial genetics suppressor screen allowed us to discover the uridyltransferase activity possessed by GlmR.

GlmR mutants in L. monocytogenes and other organisms demonstrate both cell-wall stress response defects and defects during growth on gluconeogenic substrates (18, 19, 22). Although we cannot rule out that GlmR has potentially separable functions in gluconeogenic metabolism and cell-wall precursor metabolism (22), our identification of suppressor mutations that rescue virulence through restoration of UDP-GlcNAc levels suggests that GlmR’s role in mediating cell-wall homeostasis via UDP-GlcNAc is critical during infection. GlmR’s function in promoting cytosolic survival further suggests that bacteria experience cell-wall stress in the cytosol; however, the cytosolic CAD responsible for imparting cell-wall stress is unknown. Guanylate binding proteins (GBPs) and lysozyme are not responsible for the cytosolic cell-wall stress as GlmR is required for cytosolic survival even in Gbp^Chr3−/−^ and LysM^−/−^ macrophages (16, 41). Furthermore, our group has previously published that the PASTA (penicillin binding protein and serine/threonine kinase associated) kinase cell-wall stress sensor PrkA is essential for survival in the cytosol, consistent with a direct cell-wall-acting stressor in the macrophage cytosol (42). Alternatively, the GlmR function in promoting cytosolic survival may be due to metabolic stress tied to its potential role in growth on gluconeogenic substrates such as glycerol during cytosolic replication. Importantly, a role for GlmR in direct cell-wall stress responses versus dealing with metabolic stress in promoting cytosolic survival and virulence are not mutually exclusive. Future identification of the cytosolic CADs targeting the bacterial cell wall will illuminate novel host defense pathways, not only against L. monocytogenes, but also other bacteria that invade the cytosol, including both canonical and noncanonical cytosolic pathogens such as M. tuberculosis and S. aureus. Furthermore, other bacterial pathogens that require GlmR for survival and virulence, such as S. aureus (21) and M. tuberculosis (19, 20), likely require GlmR to deal with cell-wall stress in their conventional replication niches.

We found that GlmR uridyltransferase activity is conserved in S. aureus, B. subtilis, and M. tuberculosis (data not shown), representatives of the Firmicutes and Actinobacteria phyla. This conservation, combined with its essential role in virulence of a number of important pathogens, suggests that it may be an attractive drug candidate. Indeed, both the acetyl- and uridyltransferase activities of M. tuberculosis GlmU have been targeted by small molecules as a novel antibiotic strategy (43). Whether uridyltransferase inhibitors of GlmU could also bind and inhibit GlmR will need to be assessed. Among GlmR homologues, the N-terminal putative nucleotide binding region is most highly conserved. This raises important questions not only about the design of GlmR small molecule inhibitors, but also about substrate specificity of GlmR homologues and whether different GlmR proteins may have flexibility to catalyze different reactions with regard to the sugar component. Indeed, this may explain why GlmR appears to have a role in cell-wall homeostasis and alternatively a role in gluconeogenic metabolism. Crystal structures of GlmR homologues in complex with their substrates will be critical both for antibiotic development and an understanding of the potential promiscuity of these enzymes.

GlmR uridyltransferase activity is conserved, but the mechanism(s) of regulation of GlmR expression and/or activity remains unknown. In L. monocytogenes, GlmR is upregulated at the protein level by cell-wall stress (16). Additionally, GlmR is phosphorylated by PASTA kinases in L. monocytogenes, B. subtilis, and M. tuberculosis; however, the phosphorylation sites differ, and what effect phosphorylation may have on the enzymatic activity is similarly unknown (16, 19, 44). Subcellular localization of GlmR may also contribute to its regulation as GlmR localization patterns in B. subtilis and M. tuberculosis are dissimilar but consistent with localization of peptidoglycan synthesis in these organisms (19, 22, 45, 46). Finally, recent studies have suggested that GlmR may also act allosterically to regulate the function of GlmS in B. subtilis (26, 31). Although we were unable to observe this interaction in L. monocytogenes, GlmR functioning as an allosteric regulator of GlmS and as a functional uridyltransferase are not mutually exclusive and indeed could act synergistically. Identification of mutations that abolish GlmS–GlmR interaction but not enzymatic activity and vice versa are necessary to separate and test these ideas.

This study identified that GlmR, a protein required for L. monocytogenes and M. tuberculosis virulence, is an accessory uridyltransferase necessary for UDP-GlcNAc synthesis in the context of cell-wall stress. Similar to MurA and MurZ in S. aureus (35), this highlights that virulence determinants can be redundant with essential housekeeping enzymes. Often these accessory enzymes are upregulated in the context of stress, such as during infection or antibiotic treatment as is the case with GlmR and MurZ, respectively (35). Indeed, GlmR’s enzymatic activity may have gone previously undiscovered despite its importance, because of the protein’s low expression during normal laboratory culture with rich media. Additionally, with a potential exception in S. aureus (21), GlmR is likely not essential under laboratory conditions, because of sufficient uridyltransferase activity of GlmU. Conversely, even in a situation where GlmR complemented GlmU uridyltransferase activity, GlmU would still be essential due to its acetyl-transferase function. Future analysis of virulence determinants of unknown function through parallel screening approaches may reveal this redundancy to be even more pervasive.

## MATERIALS AND METHODS

### Listeria monocytogenes strains and culture.

All L. monocytogenes strains used for experiments in this study were 10403S background. The Δ*glmR* mutant was described previously (14). L. monocytogenes was grown overnight in brain heart infusion (BHI) at 30°C for all experiments except as described for metabolomic analysis.

### Construction of L. monocytogenes strains.

Homologue complementation genes used in Fig. 4 were created with gBlocks (IDT) that were codon-optimized for L. monocytogenes and inserted into pIMK2 (47) under the control of the constitutive pHelp promoter. Constructs were cloned in XL1-Blue E. coli with 30 μg/mL kanamycin for pIMK2 and shuttled into L. monocytogenes through conjugation with SM10 or S17 E. coli.

### Suppressor selection.

A Himar 1 Tn mutant library was created in a Δ*glmR* mutant background as described previously (48). Aliquots of the library were thawed, diluted 1:1,000–10,000 in PBS, and inoculated 1:50 into 1 mL of Luria broth (LB) with 1 mg/mL lysozyme and 0.1 μM staurosporine in pentaplicate. Fifty microliters of cultures were plated at 0 h on LB and 6 h on LB 1 mg/mL lysozyme. This selection was carried out four times, and 313 out of 476 resulting colonies were secondarily screened in BHI with lysozyme 1 mg/mL and staurosporine 0.1 μM. Transposon mutations in the remaining suppressors were identified by 2-step PCR using transposon-specific and degenerate primers followed by sanger sequencing and were confirmed by PCR with diagnostic primers (49). To determine whether identified transposon mutations were causative, all unique transposons were transduced into a fresh Δ*glmR* background and reconfirmed with diagnostic PCR, sequencing, and rescue of the Δ*glmR* mutant lysozyme sensitivity with overnight growth in 1 mg/mL lysozyme in BHI.

### Phage transduction.

Phage transductions were performed as previously described (50). Briefly, U153 phage stocks were propagated with MACK L. monocytogenes grown overnight in LB at 30°C. MACK cultures were pelleted and resuspended in LB with 10 mM MgCl_2_ and 10 mM CaSO_4_, mixed with 0.7% LB agar 10 mM MgCl_2_ 10 mM CaSO_4_ at 42°C, and immediately poured on LB plates and incubated overnight at 30°C. Plaque lysate was soaked out with 10 mM Tris pH 7.5 10 mM MgCl_2_ 10 mM CaSO_4_ buffer and sterilized by 0.2 μm filtration or addition of 1:3 volume chloroform. Donor plaque lysates were prepared using the same conditions and used to infect recipient Δ*glmR* cultures for 1 h at room temperature before being plated on erythromycin selection at 37°C.

### Lysozyme sensitivity.

Overnight 30°C static BHI cultures were backdiluted 1:50 into 96-well plates containing BHI or BHI with lysozyme at 1 mg/mL. Plates were grown at 37°C with continuous shaking for 12 h in an Eon or Synergy HT Microplate Spectrophotometer (BioTek Instruments, Inc., Winooski, VT), and OD_600_ was read every 15 min.

### Plaque assay.

The plaque assay was performed as described (27) except that the multiplicity of infection (MOI) was adjusted for optimal plaque number and an additional agarose media plug was added to wells at 3 days to facilitate an additional 3 days of plaque growth. At 6 days, wells were stained with 0.3% crystal violet and washed with water. After staining, the dishes were scanned and plaque areas were quantified with ImageJ. All strains were assayed in biological triplicate, and the plaque areas of each strain were normalized to wild-type plaque size within each replicate.

### Metabolite extraction.

Overnight 30°C static BHI cultures were washed with PBS and backdiluted 1:50 into 50 mL of Listeria synthetic media (LSM) baffled flasks at 37°C, shook, and grown to an OD600 of ~0.4. LSM is a derivative of Improved Minimal Media developed by Phan-Thanh and Gorman (51) with several component changes (52). For metabolomic experiments, we reduced the level of morpholinepropanesulfonic acid (MOPS) to 1/5th the normal amount to reduce background MS signal. Five milliliters of culture were deposited by vacuum filtration onto a 0.2-μm nylon membrane (47 mm diameter) in duplicate. The membrane was then placed (cells down) into 1.5 mL cold (−20°C or on dry ice) extraction solvent (20:20:10 vol/vol/vol acetonitrile, methanol, water) in a 60-mm petri dish and swirled. After a few moments, the filter was inverted (cells up) and solvent was passed over the surface of the membrane several times to maximize extraction. Finally, the cell extract was stored at −80°C. Extracts were pelleted at 21,000 rcf (relative centrifugal force) at 4°C for 10 min. We dried ~200 μL of extract normalized to OD, with N_2_ gas. Extracts were resuspended in 70 μL of high-pressure liquid chromatography (HPLC)-grade water and pelleted at 2,1000 rcf at 4°C for 10 min to remove particulates. All cultures were extracted in biological triplicate or quadruplicate and in technical duplicate.

### Metabolite quantification and analysis.

Metabolite quantification and analysis was performed with the same instrument, and chromatography set up as previously described (53). Briefly, samples were run through an ACQUITY UPLC BEH C_18_ column in an 18-min gradient with Solvent A consisting of 97% water, 3% methanol, 10 mM tributylamine (TBA), 9.8 mM acetic acid, pH 8.2, and Solvent B being 100% methanol. Gradient was 5% Solvent B for 2.5 min, gradually increased to 95% Solvent B at 18 min, held at 95% Solvent B until 20.5 min, returned to 5% Solvent B over 0.5 min, and held at 5% Solvent B for the remaining 4 min. Ions were generated by heated electrospray ionization (HESI; negative mode) and quantified by a hybridquadrupole high-resolution mass spectrometer (Q Exactive Orbitrap, Thermo Scientific). MS scans consisted of full MS scanning for 70 to 1,000 *m/z* from time zero to18 min, except that MOPS *m/z* of 208 to 210 was excluded from 1.5 to 3 min. Metabolite peaks were identified using the Metabolomics Analysis and Visualization Engine (MAVEN) (30, 54).

### Protein purification.

**(i) GST tagged protein expression and purification scheme.** GlmR, GlmR3, and GlmU were cloned into pGex6P in XL1-Blue *E. coli.* Following confirmation by sequencing, pGex6P vectors were subsequently transformed into Rosetta *E. coli.* IPTG was added to 500 μM to induce expression, and 3 h postinduction cells were pelleted, resuspended in PBS, and frozen at –80C. Cell suspensions were thawed and lysed by sonication in the presence of protease inhibitors. Cell debris was pelleted, and cell lysate was filtered with a 0.2-μM filter and loaded onto a prepacked glutathione resin column at 4°C. The column was washed two times with 10-column volumes of cleavage buffer (25 mM Tris, pH 8, 100 mM NaCl, 1 mM DTT) before elution. The column was loaded with 80 units of PreScission Protease in 960 μL of cleavage buffer and incubated overnight at 4°C. Elution was collected the next day by adding 3 mL of cleavage buffer to the column, and concentrated between 15 μM and 23 μM. Protein was stored at 4°C, purity was assessed by SDS-Page, and protein was quantified by bicinchoninic acid BCA assay.

### His-tagged protein expression and purification scheme.

GlmR homologues were cloned into pET20b in XL1-Blue and Rosettas with pLysS except for CuvA, which was expressed from BL21(DE3) from pET23. IPTG was added to 500 μM to induce expression, and 3 h postinduction, cells were pelleted, resuspended in PBS, and frozen at −80°C. Cell suspensions were thawed and lysed by sonication in the presence of protease inhibitors. Cell debris was pelleted, and cell lysate was filtered with a 0.2-μm filter and loaded onto a HisTrap Ni column (GE Healthcare) at 4°C. The column was washed with PBS and PBS 25 mM imidizole before elution with 250 mM imidizole. Elutions were dialyzed overnight at 4°C into 10 mM Tris pH 7.4 100 mM NaCl, which was prepared at 25°C and concentrated to between 6 and 22 μM. Protein was stored at 4°C, purity was assessed by SDS-PAGE, and protein was quantified by bicinchoninic acid (BCA) assay.

### Enzymatic activity.

Reactions were carried out in 10 mM Tris pH 7.4, 100 mM NaCl, and 1 mM MgCl_2_ buffer. Substrates (GlcNAc-1-P, UTP, or Acetyl CoA) were added at 100 μM, and purified E. coli GlmU (Galen Laboratory Supplies, GL01012), L. monocytogenes GlmU, L. monocytogenes GlmR, GlmR homologues, and heat-inactivated (HI) L. monocytogenes GlmR or GlmU were added at 1 μM and incubated at 37°C for 10 min. Protein was removed with a 3-kDa molecular weight cutoff (MWCO) filter, and resulting reaction mixtures were diluted 1 to 10 in solvent A and analyzed by tandem HPLC-MS and Maven software.

### Bacterial two-hybrid.

GlmR and GlmS from both L. monocytogenes and B. subtilis were cloned in-frame into vectors pU18, pU18C, pKT25, and pKNT25 from the BACTH System kit (Euromedex) using XbaI and KpnI. Constructs were made originally in TAM1 or XL1-Blue E. coli and then moved to BTH101 E. coli for testing. Both blue/white screening on X-gal plates and β-galactosidase assays were carried out as previously described (32).

### Mouse infection.

Infections were performed as previously described (16). Briefly, 6- to 8-week-old female and male C57BL/6 mice were infected IV with 1 × 10^5^ CFU. Forty-eight hours postinfection, livers and spleens were harvested, homogenized in PBS with 0.1% NP-40, and plated for CFU. Two independent replicates of each experiment with 5 mice per group were performed.

### Ethics statement.

Mice were cared for according to the recommendations of the NIH, published in the Guide for the Care and Use of Laboratory Animals. All techniques used were reviewed and approved by the University of Wisconsin—Madison Institutional Animal Care and Use Committee (IACUC) under the protocol M005916.

### Statistical analysis.

Prism 6 (GraphPad Software) was used for statistical analysis of data. Means from two groups were compared with unpaired two-tailed Student’s *t* test. Means from more than two groups were analyzed by one-way ANOVA with a *post hoc* LSD (least significant difference) test. A Mann-Whitney test was used to analyze nonnormal data from animal experiments. * indicates a statistically significant difference (*P* is less than 0.05).

10.1128/mbio.00073-23.1 TEXT S1 Supplementary methods. Download Text S1, DOCX file, 0.01 MB. Copyright © 2023 Pensinger et al. 2023 Pensinger et al. https://creativecommons.org/licenses/by/4.0/ This content is distributed under the terms of the Creative Commons Attribution 4.0 International license.
