# Supplementary material for: Listeria monocytogenes GlmR Is an Accessory Uridyltransferase Essential for Cytosolic Survival and Virulence
Source: mBio. 2023 Mar 20;14(2):e00073-23. doi: 10.1128/mbio.00073-23 (PMC10128056; doi:10.1128/mbio.00073-23)
Supplement: TABLE S1 [file mbio.00073-23-s0008.docx]

| **Table S1– putative KEGG identified differential metabolites** | | | | | |
| --- | --- | --- | --- | --- | --- |
| **Median Retention Time** | **Median M/Z** | **Kegg predicted metabolite** | **Wild-type average** | **Δ*glmR* average** | **Ratio** |
| 9.4843 | 199.0011 | D-Erythrose 4-phosphate | 1.13E+05 | 1.12E+06 | 0.101704217 |
| 11.57196 | 261.1342 | Streptidine | 3.92E+05 | 4.36E+06 | 0.090034284 |
| 11.26841 | 332.0469 | 3-[(2-Chlorobenzylidene)amino]-6H-dibenzo[b-d]pyran-6-one | 1.77E+05 | 1.00E+06 | 0.175920064 |
| 11.79669 | 187.0976 | Azelaic acid | 3.73E+06 | 2.02E+07 | 0.184657409 |
| 12.40659 | 275.1501 | N-(4-Guanidinobutyl)-4-hydroxycinnamide | 1.72E+05 | 8.18E+05 | 0.210368222 |
| 11.80024 | 209.0794 | N4-Phosphoagmatine | 1.85E+05 | 8.45E+05 | 0.219326987 |
| 11.59127 | 157.0506 | 2-Isopropylmaleate | 5.27E+06 | 1.73E+07 | 0.304484059 |
| 10.21624 | 459.0932 | Anhydrochlortetracycline | 1.36E+05 | 4.22E+05 | 0.323036636 |
| 6.080052 | 217.0908 | 2-Oxo-9-methylthiononanoic acid | 3.62E+05 | 1.11E+06 | 0.325882086 |
| 11.88695 | 464.0982 | Delphinidin 3-O-glucoside | 2.29E+05 | 7.01E+05 | 0.326941165 |
| 6.430722 | 217.0828 | gamma-L-Glutamyl-D-alanine | 1.47E+05 | 4.48E+05 | 0.328082992 |
| 10.05765 | 181.0506 | 3-4-Dihydroxyphenylpropanoate | 3.42E+05 | 1.03E+06 | 0.332518102 |
| 10.41157 | 336.0602 | 5-Hydroxymethyldeoxycytidylate | 1.05E+05 | 2.99E+05 | 0.350062002 |
| 14.37131 | 244.0073 | Guanfacine | 6.66E+05 | 1.86E+06 | 0.358515244 |
| 1.792438 | 128.0104 | Cyanuric acid | 1.06E+05 | 2.67E+05 | 0.397410261 |
| 1.505699 | 174.0771 | 2-Benzimidazolylguanidine | 1.51E+05 | 3.71E+05 | 0.406431227 |
| 17.33747 | 277.1444 | Dibutyl phthalate | 1.92E+06 | 4.69E+06 | 0.409928425 |
| 10.79509 | 440.1136 | 9-Hydroxy-3-5-7-11-13-15-17-octaoxo-eicosanoyl-[acp] | 4.44E+05 | 1.06E+06 | 0.41821093 |
| 10.64321 | 189.0404 | 4-Hydroxy-2-oxo-heptanedioate | 8.86E+06 | 2.06E+07 | 0.430467446 |
| 10.65088 | 129.0194 | Acetylpyruvate | 1.31E+07 | 3.00E+07 | 0.43652598 |
| 1.741757 | 215.1036 | gamma-Glutamyl-gamma-aminobutyraldehyde | 4.55E+06 | 1.04E+07 | 0.438395715 |
| 14.17814 | 279.0775 | Methyl nigakinone | 8.80E+05 | 1.97E+06 | 0.446892842 |
| 9.255281 | 101.0605 | Pentanoate | 1.06E+06 | 2.35E+06 | 0.451956289 |
| 14.92188 | 312.1242 | Laurolitsine | 1.26E+05 | 2.75E+05 | 0.457112993 |
| 11.48864 | 165.0193 | 4-Formylsalicylic acid | 2.15E+06 | 4.54E+06 | 0.473787202 |
| 1.466906 | 180.0794 | Acetylcholine chloride | 1.23E+05 | 2.60E+05 | 0.47405135 |
| 7.732138 | 309.0649 | Sulfadoxine | 1.38E+05 | 2.90E+05 | 0.474682745 |
| 12.87267 | 188.9515 | 2-4-Dichlorobenzoate | 1.66E+05 | 3.37E+05 | 0.493700679 |
| 1.990167 | 284.1673 | Isococculidine | 2.01E+05 | 4.04E+05 | 0.496476805 |
| 1.121834 | 482.0749 | Peptide(N-Glu- Asp- Cystine) | 2.33E+05 | 1.11E+05 | 2.092764267 |
| 1.435915 | 266.0702 | S-Ribosyl-L-homocysteine | 2.35E+05 | 1.01E+05 | 2.323378813 |
| 11.44765 | 281.1006 | 2-Aminoadenosine | 2.83E+05 | 1.12E+05 | 2.533581026 |
| 11.50283 | 298.0697 | Avenanthramide A | 6.41E+05 | 2.49E+05 | 2.573129783 |
| 14.15145 | 276.0337 | Azathioprine | 5.97E+05 | 2.08E+05 | 2.866727237 |
| 10.14312 | 606.0742 | UDP-N-acetyl-D-glucosamine | 4.63E+07 | 1.43E+07 | 3.229520949 |
| 14.46659 | 244.0073 | Guanfacine | 8.67E+05 | 2.37E+05 | 3.650453862 |
| 9.587825 | 308.0988 | N-Acetylneuraminate | 8.56E+05 | 2.15E+05 | 3.979941319 |
