# Supplementary material for: Listeria monocytogenes GlmR Is an Accessory Uridyltransferase Essential for Cytosolic Survival and Virulence
Source: mBio. 2023 Mar 20;14(2):e00073-23. doi: 10.1128/mbio.00073-23 (PMC10128056; doi:10.1128/mbio.00073-23)
Supplement: FIG S3 [file mbio.00073-23-s0003.pdf]

A

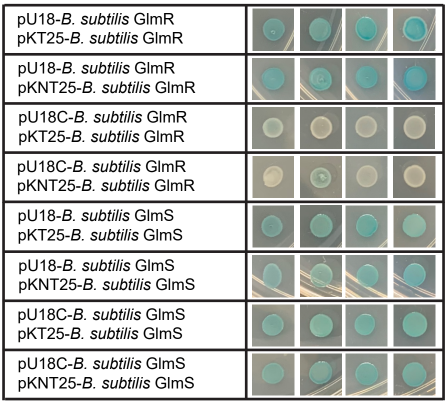
pU18-*B. subtilis* GlmR
pKT25-*B. subtilis* GlmR
pU18-*B. subtilis* GlmR
pKNT25-*B. subtilis* GlmR
pU18C-*B. subtilis* GlmR
pKT25-*B. subtilis* GlmR
pU18C-*B. subtilis* GlmR
pKNT25-*B. subtilis* GlmR
pU18-*B. subtilis* GlmS
pKT25-*B. subtilis* GlmS
pU18-*B. subtilis* GlmS
pKNT25-*B. subtilis* GlmS
pU18C-*B. subtilis* GlmS
pKT25-*B. subtilis* GlmS
pU18C-*B. subtilis* GlmS
pKNT25-*B. subtilis* GlmS

B

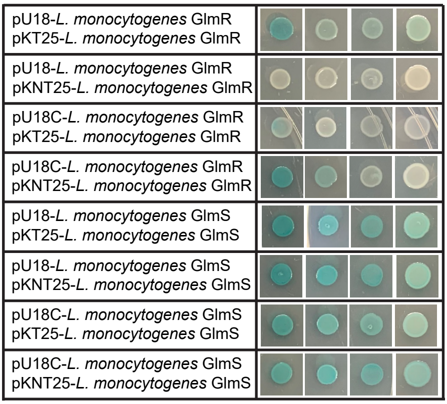
pU18-*L. monocytogenes* GlmR
pKT25-*L. monocytogenes* GlmR
pU18-*L. monocytogenes* GlmR
pKNT25-*L. monocytogenes* GlmR
pU18C-*L. monocytogenes* GlmR
pKT25-*L. monocytogenes* GlmR
pU18C-*L. monocytogenes* GlmR
pKNT25-*L. monocytogenes* GlmR
pU18-*L. monocytogenes* GlmS
pKT25-*L. monocytogenes* GlmS
pU18-*L. monocytogenes* GlmS
pKNT25-*L. monocytogenes* GlmS
pU18C-*L. monocytogenes* GlmS
pKT25-*L. monocytogenes* GlmS
pU18C-*L. monocytogenes* GlmS
pKNT25-*L. monocytogenes* GlmS
