# Supplementary material for: Listeria monocytogenes GlmR Is an Accessory Uridyltransferase Essential for Cytosolic Survival and Virulence
Source: mBio. 2023 Mar 20;14(2):e00073-23. doi: 10.1128/mbio.00073-23 (PMC10128056; doi:10.1128/mbio.00073-23)
Supplement: FIG S5 [file mbio.00073-23-s0005.pdf]

A

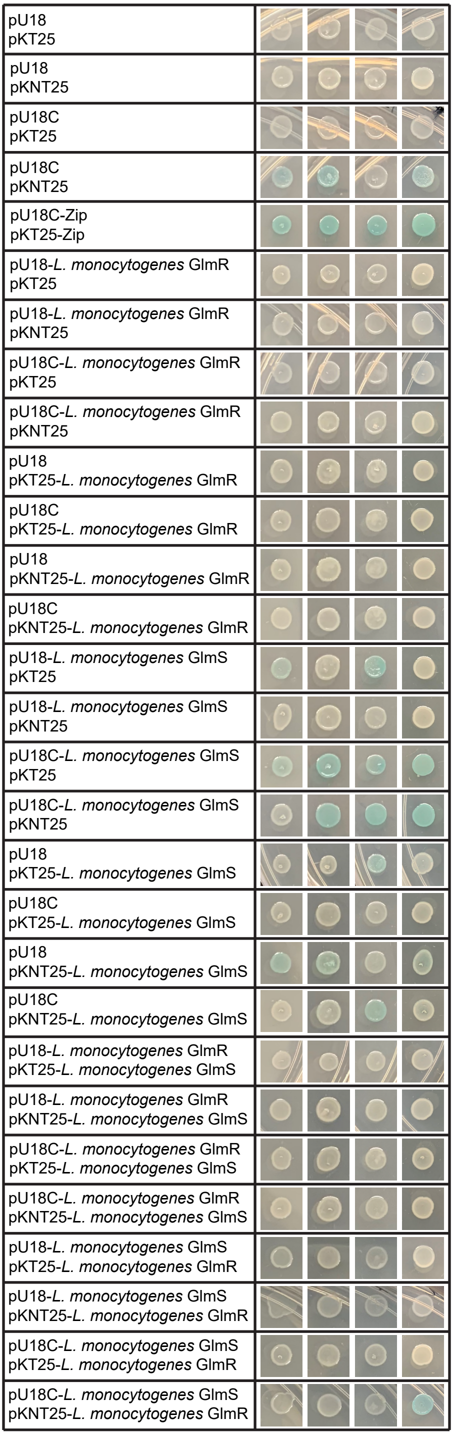

B

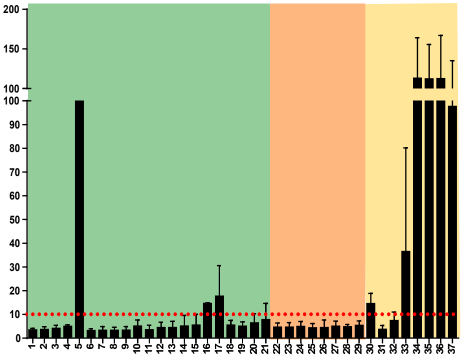

1	pU18	pKT25
2	pU18	pKNT25
3	pU18C	pKT25
4	pU18C	pKNT25
5	pU18C Zip	pKT25 Zip
6	pU18 Lm *glmR*	pKT25
7	pU18 Lm *glmR*	pKNT25
8	pU18C Lm *glmR*	pKT25
9	pU18C Lm *glmR*	pKNT25
10	pU18	pKT25 Lm *glmR*
11	pU18C	pKT25 Lm *glmR*
12	pU18	pKNT25 Lm *glmR*
13	pU18C	pKNT25 Lm *glmR*
14	pU18 Lm *glmS*	pKT25
15	pU18 Lm *glmS*	pKNT25
16	pU18C Lm *glmS*	pKT25
17	pU18C Lm *glmS*	pKNT25
18	pU18	pKT25 Lm *glmS*
19	pU18C	pKT25 Lm *glmS*
20	pU18	pKNT25 Lm *glmS*
21	pU18C	pKNT25 Lm *glmS*
22	pU18 Lm *glmR*	pKT25 Lm *glmS*
23	pU18 Lm *glmR*	pKNT25 Lm *glmS*
24	pU18C Lm *glmR*	pKT25 Lm *glmS*
25	pU18C Lm *glmR*	pKNT25 Lm *glmS*
26	pU18 Lm *glmS*	pKT25 Lm *glmR*
27	pU18 Lm *glmS*	pKNT25 Lm *glmR*
28	pU18C Lm *glmS*	pKT25 Lm *glmR*
29	pU18C Lm *glmS*	pKNT25 Lm *glmR*
30	pU18 Lm *glmR*	pKT25 Lm *glmR*
31	pU18 Lm *glmR*	pKNT25 Lm *glmR*
32	pU18C Lm *glmR*	pKT25 Lm *glmR*
33	pU18C Lm *glmR*	pKNT25 Lm *glmR*
34	pU18 Lm *glmS*	pKT25 Lm *glmS*
35	pU18 Lm *glmS*	pKNT25 Lm *glmS*
36	pU18C Lm *glmS*	pKT25 Lm *glmS*
37	pU18C Lm *glmS*	pKNT25 Lm *glmS*
