# Supplementary material for: Listeria monocytogenes GlmR Is an Accessory Uridyltransferase Essential for Cytosolic Survival and Virulence
Source: mBio. 2023 Mar 20;14(2):e00073-23. doi: 10.1128/mbio.00073-23 (PMC10128056; doi:10.1128/mbio.00073-23)
Supplement: TEXT S1 [file mbio.00073-23-s0009.docx]

**Supplementary Methods**

**Wheat Germ Agglutinin Staining**

1mL of overnight cultures in BHI at 37°C were pelleted, fixed in 4% paraformaldehyde in PBS, washed in PBS with 0.1% Tween (PBS-T), resuspended in 100μL PBS-T, and incubated with 50μL of 0.1% Wheat Germ Agglutinin (WGA) for 5 minutes. Pellets were washed in PBS-T and stored at 4°C in the dark. Confocal microscopy was performed as previously described ^48^.
