# Supplementary material for: Listeria monocytogenes GlmR Is an Accessory Uridyltransferase Essential for Cytosolic Survival and Virulence
Source: mBio. 2023 Mar 20;14(2):e00073-23. doi: 10.1128/mbio.00073-23 (PMC10128056; doi:10.1128/mbio.00073-23)
Supplement: FIG S4 [file mbio.00073-23-s0004.pdf]

A

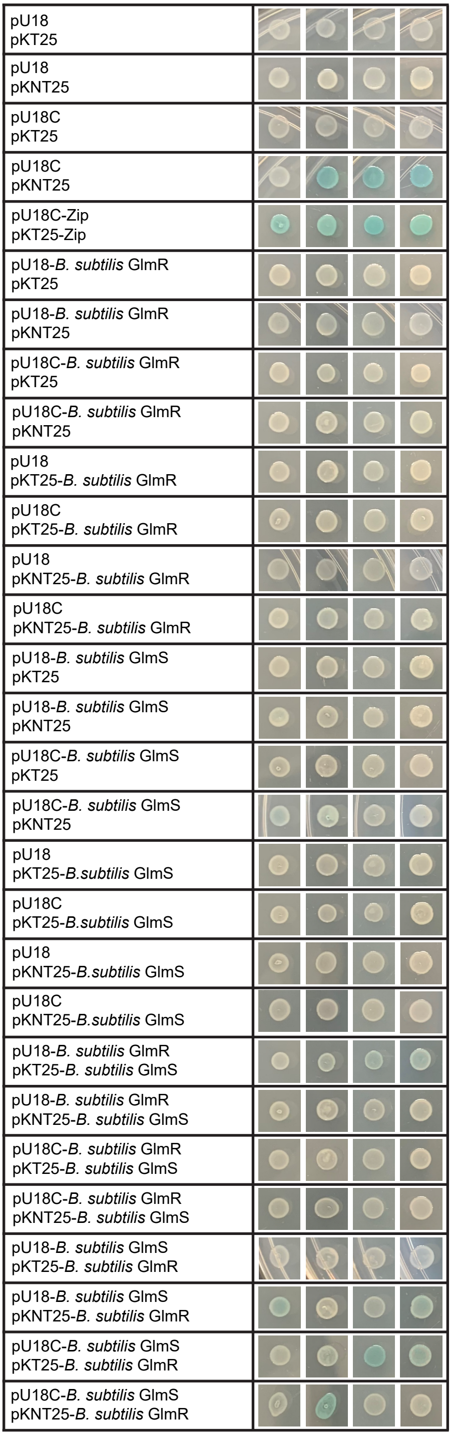

B

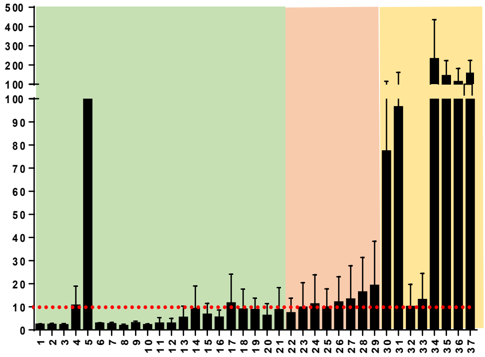

1	pU18	pKT25
2	pU18	pKNT25
3	pU18C	pKT25
4	pU18C	pKNT25
5	pU18C Zip	pKT25 Zip
6	pU18 Bs glmR	pKT25
7	pU18 Bs glmR	pKNT25
8	pU18C Bs glmR	pKT25
9	pU18C Bs glmR	pKNT25
10	pU18	pKT25 Bs glmR
11	pU18C	pKT25 Bs glmR
12	pU18	pKNT25 Bs glmR
13	pU18C	pKNT25 Bs glmR
14	pU18 Bs glmS	pKT25
15	pU18 Bs glmS	pKNT25
16	pU18C Bs glmS	pKT25
17	pU18C Bs glmS	pKNT25
18	pU18	pKT25 Bs glmS
19	pU18C	pKT25 Bs glmS
20	pU18	pKNT25 Bs glmS
21	pU18C	pKNT25 Bs glmS
22	pU18 Bs glmR	pKT25 Bs glmS
23	pU18 Bs glmR	pKNT25 Bs glmS
24	pU18C Bs glmR	pKT25 Bs glmS
25	pU18C Bs glmR	pKNT25 Bs glmS
26	pU18 Bs glmS	pKT25 Bs glmR
27	pU18 Bs glmS	pKNT25 Bs glmR
28	pU18C Bs glmS	pKT25 Bs glmR
29	pU18C Bs glmS	pKNT25 Bs glmR
30	pU18 Bs glmR	pKT25 Bs glmR
31	pU18 Bs glmR	pKNT25 Bs glmR
32	pU18C Bs glmR	pKT25 Bs glmR
33	pU18C Bs glmR	pKNT25 Bs glmR
34	pU18 Bs glmS	pKT25 Bs glmS
35	pU18 Bs glmS	pKNT25 Bs glmS
36	pU18C Bs glmS	pKT25 Bs glmS
37	pU18C Bs glmS	pKNT25 Bs glmS
